# Editorial: The Role of the Individual in the Great Transformation Toward Sustainability

**DOI:** 10.3389/fpsyg.2021.710897

**Published:** 2021-07-05

**Authors:** Sebastian Bamberg, Daniel Fischer, Sonja M. Geiger

**Affiliations:** ^1^Department for Social Sciences, Bielefeld University of Applied Sciences, Bielefeld, Germany; ^2^Department for Social Sciences, Wageningen University and Research, Wageningen, Netherlands; ^3^Department of Consumer Research, Communication and Food Sociology, Justus Liebig University, Giessen, Germany

**Keywords:** socio-ecological transformation, sustainable development, environmental psychology, education for sustainable development, multi-level perspective framework, research avenues

Natural scientists have repeatedly cautioned that various planetary boundaries that are thought to safeguard earth's systems equilibrium have been trespassed in recent years (Steffen et al., 2015). At the same time, many people are still denied the opportunity to satisfy their basic needs (UNDP, 2020). The question of how to guide human development on this planet on a just and safe trajectory has therefore become a central task of our time (Häyhä et al., 2016; O'Neill et al., 2018). Science is expected to make important contributions to this task (Bai et al., 2016). In recent years, it has become clear that technological innovations alone will not be enough, but that changes in human behavior are needed on a large scale (Wiedmann et al., 2020; Newell et al., 2021). Why has the human response to the multiple social and environmental crises been so inadequate despite the growing global concern about their consequences? Why are people not taking the right measures to advance sustainable development? Providing insights that help to answer these questions should be the central task for both psychologists and educational scientists concerned with human behavior and learning.

However, we have to confess that the research results currently produced by mainstream psychology and the learning sciences provide little politically impactful insights to answer this question. Why has psychology—a science devoted to the understanding and prediction of human behavior—such difficulties to answer the question “Why aren't people taking enough action?” Why have questions like “How can we facilitate individual and social learning for change toward sustainability?” received so little attention in the mainstream of the education and the learning sciences so far? The desire to deal more systematically with this uncomfortable issue has provided the motivation for organizing this Research Topic. As the editorial team, we share the assumption that answers to the above raised questions have to start with a critique of the problematic meta-theoretical perspective currently underlying mainstream psychological and educational research on sustainability. According to this problematic meta-theoretical perspective, at the core of unsustainable developments lie the wrong decisions of billions of individual consumers. Consequently, the central task of psychological and educational research consists in understanding and changing the psycho-social factors or competencies that motivate the individual to change their consumption and lifestyle related choices. With this problem framing, psychology, and education alike locate the barriers preventing people from behaving in accordance to sustainability principles at the individual level rather than the social-contextual level. Schmitt et al. (2020) refer to this perspective as the “psychological barriers explanation” for inaction, a view that other scholars criticize for one-sidedly attributing responsibility to individuals and thereby ignoring and de-politicizing structural issues (“responsibilization,” see Giesler and Veresiu, 2014).

Indeed, one consequence of this deficient meta-theoretical perspective is that most psychologists and educational researchers, while in principle acknowledging their role, treat structural barriers as something separate from the psychological and learning processes they are dealing with. This is reflected in the underdeveloped theorizing of how social-structural realities might inform their analyses (for a related debate on transgressive, transformative, civic and social learning to expand an individualistic scope in environmental and sustainability education research; see Lotz-Sisitka et al., 2015; Khoo and Jørgensen, 2021). This is problematic, because treating structural barriers as separate from psychology and education denies the reality that psychological and learning processes exist within these external structures and intergroup relationships, and both shape and are shaped by them (see Reicher et al., 2012). We argue that decoupling psychological barriers to sustainable behavior from the larger systems in which these psychological processes take place, constitutes a form of psychological reductionism in which explanations for human behavior primarily consider individual mental states (Martin-Baro, 1994). It also runs the risk of promoting an instrumental form of “educationalization” that tasks educational institutions to solve societal problems by instilling pre-determined attitudes, knowledges, and behaviors (Bridges, 2008).

Furthermore, because they treat structural barriers as something separate from the psychological and pedagogical processes they are interested in, mainstream psychologists and educators tend to neglect the issues of power, inequality, and social structure. This is precisely what also makes it difficult for them to see the group-based nature of sustainability action: Not humans in general are inactive, on the contrary, many people and groups are working extremely hard for a more sustainable development of our societies. However, these people and organizations do not tend to hold much power in our societies. Others, particularly those in positions of power, such as the fossil fuel and car industry (Leonard, 2019), actively try to delay change and maintain the–unsustainable-status quo that profits them, despite environmental and human costs (Lamb et al., 2020). Thus, much of mainstream sustainability research in psychology and education neglects to raise questions about the failure of democratic institutions, how power is distributed, and why people in positions of power choose to use that power in particular ways (Fuchs et al., 2016).

In the past decade, the field of sustainability science in particular has driven efforts to understand and address sustainability not just as a thematic challenge, but also as a structural and systemic one. In the paradigm of transition management for example, the necessary changes are no longer examined primarily as those of individual behavior and individual competencies, but as transformations of layered socio-technical systems in which the individual is embedded. We are convinced that such a perspective also offers an important opportunity for psychology and education to evolve and become more effective in their own contributions to sustainable development.

To incorporate such a socio-technical systems view, we are proposing to use the multilevel perspective (MLP) developed by Geels (2002, 2004, 2011) as an integrative frame model. The MLP views societal change as occurring through the transformation of socio-technical systems. The term socio-technical indicates the complexity of such systems: They include technology, production capacities, supply networks, infrastructure, maintenance networks, legal regulation, cultural meaning, as well as user practices and markets (Geels, 2002). As the name implies, the MLP posits three analytical and heuristic levels on which processes interact and align to result in socio-technical system transformations:

(1) The landscape (macro level) describes exogenous developments such as the development of deep-seated cultural patterns, macro-politics, and economics or natural disasters. An example is climate change, but also economic crises, political upheaval, and other natural disasters (e.g., floods, droughts).

(2) Regimes (meso level) represent the current structures such as dominant rules, institutions, and technologies that are self-reinforcing. The sociotechnical regime is dynamically stable along a predictable trajectory. Many products and industries are currently based on fossil fuels, and rules and institutions were developed for these industries. This makes the regime “locked-in” and resistant to both technological and social innovations toward sustainability.

(3) Niches (micro level) are the locus for radical innovations. Incubated from market and regulation influences, the niche fosters innovations that differ fundamentally from the prevailing regime and usually require landscape developments that open windows of opportunity on the regime level. Examples in the context of the climate crisis are people who pioneer innovations as producers and investors (e.g., alternatives to fossil fuels) and citizens and activists who call for new regulations and lifestyles.

The multilevel perspective argues that transformations of socio-technical systems come about through interactions between processes at these three levels:

(a) niche innovations build up an internal momentum, through learning processes, price/performance improvements, and support from powerful groups,

(b) changes at the landscape level create pressure on the regime, and

(c) destabilization of the regime creates windows of opportunity for niche innovations.

The alignment of these processes enables the breakthrough of novelties in mainstream markets where they compete with the existing regime. Figure 1 has become a somewhat iconic picture of this dynamic.

**Figure 1:**
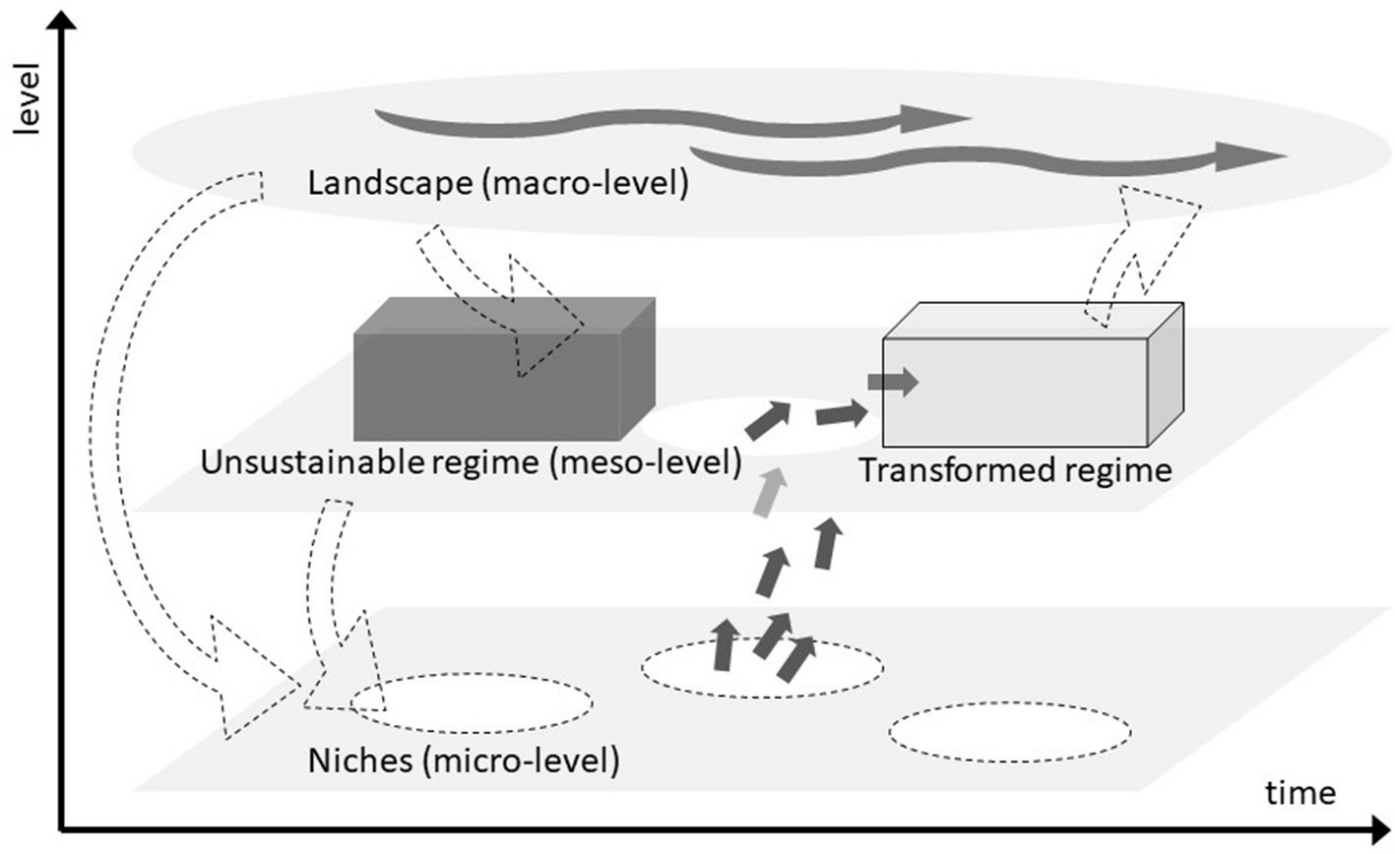
Interactions of different levels in the transformation of socio-technical systems (adapted from Geels, 2004).

What might be the practical consequences of such a paradigmatic change for sustainability-related psychological and educational research and an according adoption of a MLP perspective?

With our call for papers, we invited colleagues also interested in developing the transformative paradigm within psychology and the learning sciences to present their ideas how to theoretically relate psychological constructs and learning processes with broader social-structural contexts they are embedded in. In the following, we discuss three overarching implications of a shift toward a multi-level perspective that the contributions to this special section propose as future research avenues for psychological and education sustainability research.

First, psychological and educational researchers are challenged to broaden their traditional focus on individual, often low-impact consumption behavior toward more sustainably-relevant high impact actions in a first instance (Bilharz and Schmitt, 2011; see also current debate Nielsen et al., 2021, and van Valkengoed et al., 2021). Focusing on high-impact behaviors alone should be surpassed by opening up for peoples' role as active members of communities, citizens, and activists trying to bring about social change through their engagement in public protest and social movements. This comprises further outcome categories such as peoples' perception of environmental crises as the result of changing social discourses, their support of transformative policy alternatives as well as their decision to vote the representatives of such policies. As an example, Loy et al. focus on an individual behavior with a high environmental impact (flying) and show how psychological variables such as global identity also influence support of policy measures toward a sustainable transformation of the transport sector. In an educational perspective, Francesconi et al. describe Fridays for Future as an enactive network that acts as an informal learning space by transforming scientific knowledge to spur collective agency. Hamann et al. examine student-led sustainability initiatives as an example of how group-based coaching processes can build individual and collective efficacy that can have transformative impact in educational institutions and the communities in which they are embedded.

Second, in a sustainability context, psychologists and educational researchers alike face a world where the psychological and learning processes they are interested in exist within larger societal structures and intergroup relationships, and both shape and are shaped by them. Or more precisely, if psychologists and educational researchers want to actively contribute to the necessary transformation of socio-technical systems, they have to embed their research into a broader theoretical framework explicitly dealing with the nature and the course of societal transformation processes. Examples for this explicit consideration of structural conditions are offered by Ruhrort and Allert who focus on integrating sociological practice theory with psychological perspectives, or by Dreyer et al. who explore how structural changes can unfold cultural impacts on an organizational level. Before the backdrop of the MLP as integrative framework one central task of psychological research consists in identifying/developing transformative theoretical constructs allowing to link transformative societal processes with change oriented psychological processes. The commentary by Wullenkord and Hamann, in which the authors call for embedding existing psychological constructs in transformative theories, shows that such an embedding of psychological perspectives can indeed have innovative potential for transformation theories. Another implication of a more embedded and interactional paradigm is to better understand and account for the dynamic interaction of behavior and structure, which can sometimes also result in detrimental effects (systematic rebounds) when gains in one behavioral domain can cause new pressures on other levels (Dütschke et al.).

Third, psychology and educational science need to assert more proactively and explicitly that they are more than mere “implementation agents” tasked to achieve certain strategic goals by organizing acceptance and changing targeted behaviors. The idea of sustainability in and of itself raises normative questions about purpose: what is the envisioned state that should orient our actions and changes as “sustainable?” These questions cannot be answered technically or scientifically alone—they require deliberation. Psychological and educational contributions are needed to create conditions in the first place that empower people and enable them to participate in processes of goal clarifications, in challenging deeply ingrained worldviews and inherited patterns of thinking, and in extending the imaginary of what sustainability might mean. Supporting such “emancipatory” processes in a kind of “enabling function” is a contribution in the primarily instrumental approach to psychology and education that has received comparatively little attention so far. The contributions by Majer et al. on negotiation as well as by Bruhn on the potential contributions of therapeutic approaches indicate the direction in which work in this perspective could go.

In the end, the oeuvre of approaches to which a systemic and multilevel perspective may lead psychological and educational research must necessarily remain exemplary and incomplete at this point. The contributions to this Research Topic, which have predominantly been submitted from German-speaking research hubs, have given first answers to our questions raised and will hopefully spark a wider discussion on the future of sustainability-oriented psychological and educational research in the years to come. For the important impulses to intensify efforts in this promising avenue for future research, we are indebted to all contributing authors as well as all critical reviewers.

## Author Contributions

SB provided the first draft. DF and SG extended the draft by further aspects and edited the final version. All authors contributed to the article and approved the submitted version.

## Conflict of Interest

The authors declare that the research was conducted in the absence of any commercial or financial relationships that could be construed as a potential conflict of interest.
